# Inequities in birth registration, violent discipline, and child labour by disability status and sex: Evidence from the Multiple Indicator Cluster Surveys in 24 countries

**DOI:** 10.1371/journal.pgph.0001827

**Published:** 2023-05-24

**Authors:** Amiya Bhatia, Calum Davey, Tess Bright, Sara Rotenberg, Emily Eldred, Claudia Cappa, Hannah Kuper, Karen Devries

**Affiliations:** 1 Department of Global Health and Development, London School of Hygiene and Tropical Medicine, London, United Kingdom; 2 Faculty of Epidemiology and Population Health, London School of Hygiene and Tropical Medicine, London, United Kingdom; 3 Nuffield Department of Primary Care Health Sciences, University of Oxford, Radcliffe Observatory Quarter, Oxford, United Kingdom; 4 Data and Analytics Section, UNICEF, New York, NY, United States of America; Conservatoire national des arts et metiers, FRANCE

## Abstract

Nearly 240 million children are estimated to have a disability globally. We describe inequities by disability status and sex in birth registration, child labour, and violent discipline outcomes. Data come from Round 6 of the Multiple Indicator Cluster Survey programme and includes 323,436 children, aged 2–17 years in 24 countries. We estimated non-registration of birth, child labour, and violent discipline, stratified by sex and disability in each country. We estimated age-adjusted prevalence ratios and prevalence differences, accounting for survey design, to calculate inequities by disability. There was large variation across countries in the percentage of children with disabilities (range: 4% to 28%), in non-registration (range: 0% to73%), child labour (range: 2% to 40%), and violent discipline (range: 48% to 95%). We found relative inequities by disability in birth registration in two countries among girls and one country among boys, and in birth certification in two countries among girls and among boys. Child labour was higher among girls with disabilities in two countries and among boys in three countries. We found larger and more prevalent inequities by disability in hazardous labour in six countries among girls (aPR range: 1.23 to 1.95) and in seven countries among boys (aPR range: 1.24 to 1.80). Inequities in the prevalence of violent discipline by disability were significant in four countries among girls (aPR range: 1.02 to 1.18) and among boys (aPRs: 1.02 to 1.15) and we found inequities in severe punishment nine countries among girls (aPR range: 1.12 to 2.27) and in 13 countries among boys (aPRs: 1.13 to 1.95). Context specific research is needed to understand the large variations in inequities by disability status and sex within and across countries. Monitoring inequities in child rights by disability status and sex is important to achieve the SDGs and ensure child protection programs reduce inequities.

## Introduction

Birth registration, child labour, and violent discipline are child rights and child protection issues affecting the short- and long-term wellbeing, physical and mental health, and educational outcomes of children [1–3]. Sustainable Development Goals (SDGs) 16 (birth registration and violence) and 8 (child labour) include a commitment to addressing these issues for children [4]. Prior research has revealed inequities in birth registration, child labour, and violent discipline by sex, wealth and urban/rural residence [5, 6]. However, there remains a paucity of comparative data, particularly in low- and middle-income countries (LMICs), examining whether children with disabilities experience these outcomes more than children without disabilities, and how other factors–sex and country context—interact with disability status.

Worldwide around 240 million children have a disability [7]. Yet, children with disabilities are often missing, or invisible, in public health data [8, 9]. Existing data are also limited by measures of disability which are not comparable, and often have not included children. Existing studies on birth registration, child labour and violent discipline often lack a focus on inequities by disability, and when disability is examined, intersections with sex and country context remain unexplored. For example, few studies exist on inequities in birth registration by disability status. The limited available evidence shows that children with disabilities are more likely be in child labour [7, 10, 11], and approximately twice as likely to experience violence compared to children without disabilities, with a higher prevalence of violence in low-and middle-income countries (LMICs) [12]. A higher prevalence of child labour and violence among children with disabilities is unfair, unjust, and preventable, and constitutes a health inequity [13, 14].

The recent inclusion of the Washington Group/UNICEF Child Functioning Module (CFM) in the Multiple Indicator Cluster Survey (MICS) has generated some of the first standardised and internationally comparable nationally representative estimates of child disability and child functioning in many LMICs, where 80% of people with disabilities live [15]. A recent UNICEF report pooled these data to document inequities in child protection outcomes by disability [7]. We build on this analysis to examine how birth registration, child labour, and violent discipline outcomes are unequally distributed in each of 24 countries by disability among girls and boys.

## Methods

### Data and design

Data come from Round 6 of the UNICEF-supported MICS conducted between 2017 and 2019. MICS is a cross-sectional, household survey programme designed by UNICEF to help countries fill data gaps across several indicators of wellbeing for children and their families. Surveys are conducted by national statistical authorities (or other relevant national stakeholders), with financial and technical assistance from UNICEF [16]. The MICS use a multi-stage sampling approach to generate estimates representative at the national, regional and urban-rural level and is one of the primary data sources to monitor progress on the SDGs. Trained data collectors conduct interviews with mothers or primary caregivers about their children using standardised questionnaires. We selected MICS surveys which included data on our variables of interest and which were publicly available on the UNICEF website [16] in April 2021.

### Disability

Children with disabilities are identified using the Child Functioning Module (CFM), a module developed and validated by UNICEF and the Washington Group on Disability Statistics [17]. Questions elicit mothers’ or primary caregivers’ assessment of their children’s functional difficulties across 8 domains for children aged 24 to 59 months (seeing, hearing, mobility, fine motor, communication/comprehension, controlling behaviour and learning) and 12 domains for children aged 5–17 years (seeing, hearing, mobility, communication/comprehension, playing, controlling behaviour, learning, self-care, relationships, attention and concentrating, coping with change, affect, i.e. depression and anxiety). Response options for difficulties in controlling behaviours for children aged 24 to 59 months were “A lot more”; “More”; “The same”;“Less”; or “Not at all”. Response options for depression and anxiety were “daily”; “weekly”; “monthly”; “a few times a year” or “never”. For all the other domains, response options were “no difficulty”; “some difficulty”; “a lot of difficulty” or “cannot do at all”.

Children whose mothers or primary caregivers reported: 1) “a lot of difficulty” or “cannot do at all” on any of the functional domains or “a lot more” for controlling behaviours in children aged 24 to 59 months or 2) that a child was very sad/depressed or very anxious “daily” were considered as children with disabilities, as per UNICEF’s and Washington Group on Disability Statistics’ guidelines [18].

### Birth registration, child labour, violent discipline

Table 1 outlines the definitions used to calculate the indicators. We restricted our analysis to the ages where data were available for both the child protection outcomes and Child Functioning Module (no birth registration, 2–4 years; child labour, 5–17 years; violent discipline, 1–14 years).

**Table 1:** Definitions of child protection and disability indicators.

Indicators (caregiver reported)	Definition
No birth registration	% of children aged 2–4 years whose births were not registered
No birth certificate	% of children aged 2–4 years who did not have a birth certificate
Child labour	% of children aged 5–17 years who performed household chores or economic activities over the age-specified thresholds in the past week.
	**Economic activities** include: 1) working on a plot, farm, food, garden or looking after animals; 2) helping in a family business or a relative’s business with or without pay, or running own business; 3) producing or selling articles, handicrafts, clothes, food or agricultural products; 4) engaging in any other activity in return for income in cash or in kind, even for only one hour. **Age-specific thresholds include**: 1 hour or more in the past week for children aged 5–11 years; 14 hours or more for children aged 12–14 years; and, 43 hours or more for children aged 15–17 years.
	**Household chores** includes any of the following: 1) fetching water for household use; 2) collecting firewood for household use; 3) shopping for the household; 4) cooking; 5) washing dishes or cleaning around the house; 6) washing clothes; 7) caring for children; 8) caring for someone old or sick; 9) other household tasks. **Age-specific thresholds include:** 21 hours or more in the past week for children aged 5–11 and age 12–14 years. No threshold for children aged 15–17 years.
Hazardous labour	% of children aged 5–17 years who were engaged in hazardous working conditions in the past week.
	Hazardous labour includes work that involves: 1) carrying heavy loads; 2) working with dangerous tools such as knives and similar or operating heavy machinery. Or any exposure to: 1) dust, fumes or gas; 2) extreme cold, heat or humidity; 3) loud noise or vibration; 4) work at heights; 5) work with chemicals, such as pesticides, glues and similar, or explosives; 6) exposed to other things, processes or conditions bad for (his/her) health or safety.
Violent discipline	% of children aged 1–14 years who experienced at least one form of psychological aggression or physical punishment or severe from any adult in the household during the last month
	**Psychological aggression** included two items: 1) shouted, yelled at or screamed; 2) called them dumb, lazy or another name like that.
	**Physical punishment** included: 1) shaking; 2) spanking, hitting or slapping on the bottom with bare hand; 3) hitting on the bottom or elsewhere on the body with something like a belt, hairbrush, stick or other hard object; 4) hitting or slapped on the face, head or ears; 5) hitting or slapping on the hand, arm, or leg; 6) beating up, that is hit him/her over and over as hard as one could.
Severe punishment	% of children aged 1–14 years who experienced at least one form of severe physical punishment during the last month. This included any of: 1) hitting or slapped on the face, head or ears; 2) beating up, that is, hit him/her over and over as hard as one could.
Disability	% of children aged 2–17 years whose mothers or primary caregivers reported: 1) “a lot of difficulty” or “cannot do at all” on any of the functional domains or “a lot more” for controlling behaviours in children aged 24 to 59 months; or 2) that a child was very sad/depressed or very anxious “daily”

We included a primary measure and a more restrictive measure for each child protection indicator. For example: 1) no birth registration included all children without birth registration while no birth certificate only included children without proof of registration; 2) child labour included engagement in household chores or economic activities above an age/hours threshold in the past week while hazardous work included any dangerous work exposures (e.g., carrying heavy loads, working with dangerous tools or machinery, exposure to extreme temperatures, noise, or chemicals); 3) violent discipline included all acts of psychological or physical discipline in the past month while severe punishment included only the most severe physical acts (hitting or slapping on the face, head or ears, or beating up; hitting over and over as hard as one could). We constructed binary variables for each outcome. If data were missing from any of the items used to construct each indicator, these children were excluded from the numerator and denominator of the indicator. Across all surveys, missing data for disability (0.5%) and child protection outcomes were low (<0.2%).

### Statistical analysis

We estimated the overall and sex-stratified percentage of children with disabilities, and the prevalence of non-registration, child labour and violent discipline in each country to examine the intersections of country context, sex and disability. Prevalence estimates were cross-checked with estimates reported in survey reports and any discrepancies were noted.

We then disaggregated the prevalence of birth registration, child labour and violent discipline by disability status among boys and girls separately. We used these disaggregated prevalence estimates to calculate country-specific absolute and relative measures of inequity to examine whether children, girls, and boys with disabilities experienced a higher burden of child protection violations. We modelled the probability of the outcome for different groups with a modified Poisson model [19], accounting for the survey design and weighting using the “survey” package in R [20].

We followed guidance on health inequity measurement [21–24] and estimate both absolute and relative measures of inequities in these outcomes by disability for girls and boys separately, while also reporting the overall national prevalence and sex-stratified prevalence of each outcome. Our primary inequity measure was an age-adjusted prevalence ratio (aPR). The aPR was estimated for boys and girls in each country using the modified Poisson model, accounting for the survey design, weighting, and with robust standard errors. We assessed statistical significance of aPRs based on 95% confidence intervals. To describe the absolute magnitude of the inequity between two groups and the effort needed to close the gap, we used age-standardised prevalences [25] to calculate the prevalence difference or prevalence gap, and differences greater than 5%-points were taken to suggest a prevalence gap. We drew on the equiplot [26] to visualise overall prevalence and absolute inequities among boys and girls. We present all estimates in figures and tables, but describe and report on all estimates based on at least 30 participants in each cell in the text. All statistical analysis were done using R version 4.0.5.

### Ethical approval

The London School of Hygiene & Tropical Medicine Research Ethics Committee approved this project (Ref 22719). The MICS team in each country was responsible for seeking participant consent. All data on children is caregiver reported.

## Results

### Sample

Our sample included 323,436 children aged 2–17 years in 24 countries across 6 regions (Table 2). Half of the countries included (n = 12) were in Sub Saharan Africa, while the remainder were in Middle East and North Africa (n = 3 countries), Europe and Central Asia (n = 4), Latin America and the Caribbean (n = 2) and South Asia (n = 1; Bangladesh). Sample sizes by country ranged from 857 in Tonga to 44,420 in Bangladesh.

**Table 2:** Prevalence of disability birth registration child labour, violent discipline in 24 countries.

Countries	Any functional impairment (2–17 years)	No birth registration (2–4 years)	No birth certificate (2–4 years)	Child labour (5–17 years)	Hazardous child labour (5–17 years)	Any violent discipline (2–14 years)	Severe physical punishment (2–14 years)
**EAP**							
Mongolia	514/11,199 (5%)	1/3,796 (0%)*	18/3,796 (1%)	817/7,314 (11%)	610/7,331 (8%)	4,866/9,941 (49%)	501/9,976 (5%)
Tonga	216/2,485 (9%)	14/859 (2%)*	64/857 (7%)	456/1,623 (28%)	489/1,621 (30%)	1,869/2,086 (90%)	516/2,086 (25%)
**ECA**							
Kosovo	217/3,280 (8%)	10/923 (1%)*	85/921 (9%)	77/2,345 (3%)	122/2,347 (5%)	1,916/2,626 (73%)	154/2,623 (6%)
Kyrgyzstan	328/6,057 (7%)	9/2,167 (0%)*	21/2,167 (1%)	695/3,889 (18%)	419/3,889 (11%)	3,946/5,356 (74%)	285/5,356 (5%)
North Macedonia	172/2,364 (9%)	0/936 (0%)*	2/936 (0%)	46/1,428 (3%)	46/1,428 (3%)	1,571/2,077 (76%)	139/2,076 (7%)
Serbia	99/2,890 (4%)	1/1,163 (0%)*	12/1,162 (1%)	168/1,726 (10%)	37/1,726 (3%)	1,229/2,573 (48%)	9/2,574 (0%)
**LAC**							
Guyana	613/4,895 (15%)	23/1,692 (1%)*	105/1,691 (6%)	209/3,182 (7%)	324/3,186 (10%)	3,175/4,193 (76%)	310/4,198 (7%)
Suriname	627/6,584 (11%)	20/2,697 (0%)*	77/2,696 (3%)	121/3,867 (3%)	101/3,873 (3%)	5,052/5,771 (88%)	416/5,762 (7%)
**MENA**							
Algeria	3,694/25,605 (17%)	37/9,070 (0%)*	94/9,070 (1%)	235/16,243 (1%)	348/16,374 (2%)	18,846/22,021 (86%)	3,828/22,011 (17%)
Iraq	3,675/25,730 (18%)	56/10,162 (0%)*	380/10,145 (3%)	449/15,486 (3%)	824/15,523 (5%)	18,513/22,493 (82%)	7,600/22,484 (34%)
State of Palestine	872/9,028 (12%)	13/3,704 (0%)*	22/3,704 (0%)	323/5,251 (6%)	274/5,264 (5%)	7,117/7,869 (90%)	1,506/7,856 (21%)
**SA**							
Bangladesh	3,357/53,532 (7%)	5,173/14,048 (37%)	7,229/14,041 (51%)	2,178/39,265 (6%)	3,116/39,284 (8%)	39,542/44,420 (89%)	13,597/44,418 (31%)
**SSA**							
CAR	2,564/11,458 (27%)	2,786/5,430 (51%)	3,453/5,430 (64%)	1,431/5,972 (24%)	1,753/5,960 (29%)	9,604/10,564 (91%)	3,607/10,554 (34%)
Chad	5,928/28,821 (24%)	10,476/14,141 (74%)	10,804/14,141 (76%)	4,722/14,608 (32%)	5,853/14,629 (40%)	22,670/26,423 (86%)	7,785/26,464 (29%)
DRC Congo	4,013/26,746 (16%)	8,877/12,745 (70%)	9,469/12,745 (74%)	1,843/13,941 (13%)	2,309/13,946 (17%)	21,943/24,448 (90%)	9,308/24,423 (38%)
Ghana	2,323/14,330 (18%)	1,381/5,349 (26%)	1,784/5,337 (33%)	1,469/8,927 (16%)	1,761/8,925 (20%)	12,044/12,720 (95%)	2,240/12,721 (18%)
Guinea-Bissau	978/10,438 (14%)	2,218/4,602 (48%)	2,688/4,602 (58%)	959/5,835 (16%)	1,629/5,836 (28%)	7,558/9,454 (80%)	1,900/9,454 (20%)
Lesotho	552/7,016 (8%)	1,053/2,008 (52%)	1,176/2,004 (59%)	755/4,918 (15%)	611/4,921 (12%)	4,564/5,905 (77%)	413/5,896 (7%)
Madagascar	2,314/18,956 (13%)	1,372/7,021 (20%)	2,397/7,021 (31%)	3,745/11,915 (31%)	3,749/11,915 (31%)	14,525/16,609 (87%)	1,595/16,609 (10%)
Sao Tome and Principe	537/3,403 (18%)	14/1,212 (1%)	20/1,212 (1%)	182/2,165 (8%)	312/2,167 (14%)	2,468/2,921 (84%)	380/2,917 (13%)
Sierra Leone	3,080/18,061 (19%)	1,215/7,077 (17%)	3,125/7,053 (44%)	2,776/10,863 (26%)	3,691/10,866 (34%)	14,078/16,146 (87%)	3,973/16,126 (25%)
The Gambia	934/11,859 (9%)	2,073/6,137 (34%)	2,620/6,132 (43%)	924/5,649 (16%)	921/5,641 (16%)	9,875/10,909 (91%)	1,928/10,898 (17%)
Togo	1,211/7,913 (18%)	433/2,980 (15%)	562/2,980 (19%)	1,594/4,917 (32%)	1,489/4,917 (30%)	6,529/7,029 (93%)	1,414/7,027 (20%)
Zimbabwe	788/10,786 (9%)	1,592/3,747 (42%)	1,716/3,747 (46%)	1,672/7,005 (24%)	835/7,006 (12%)	6,202/9,467 (66%)	622/9,468 (7%)

Notes: * indicate countries that have achieved universal birth registration (<3% of children are not registered).

### Percentage of children with disabilities and prevalence of birth registration, child labour and violent discipline

**Fig 1:**
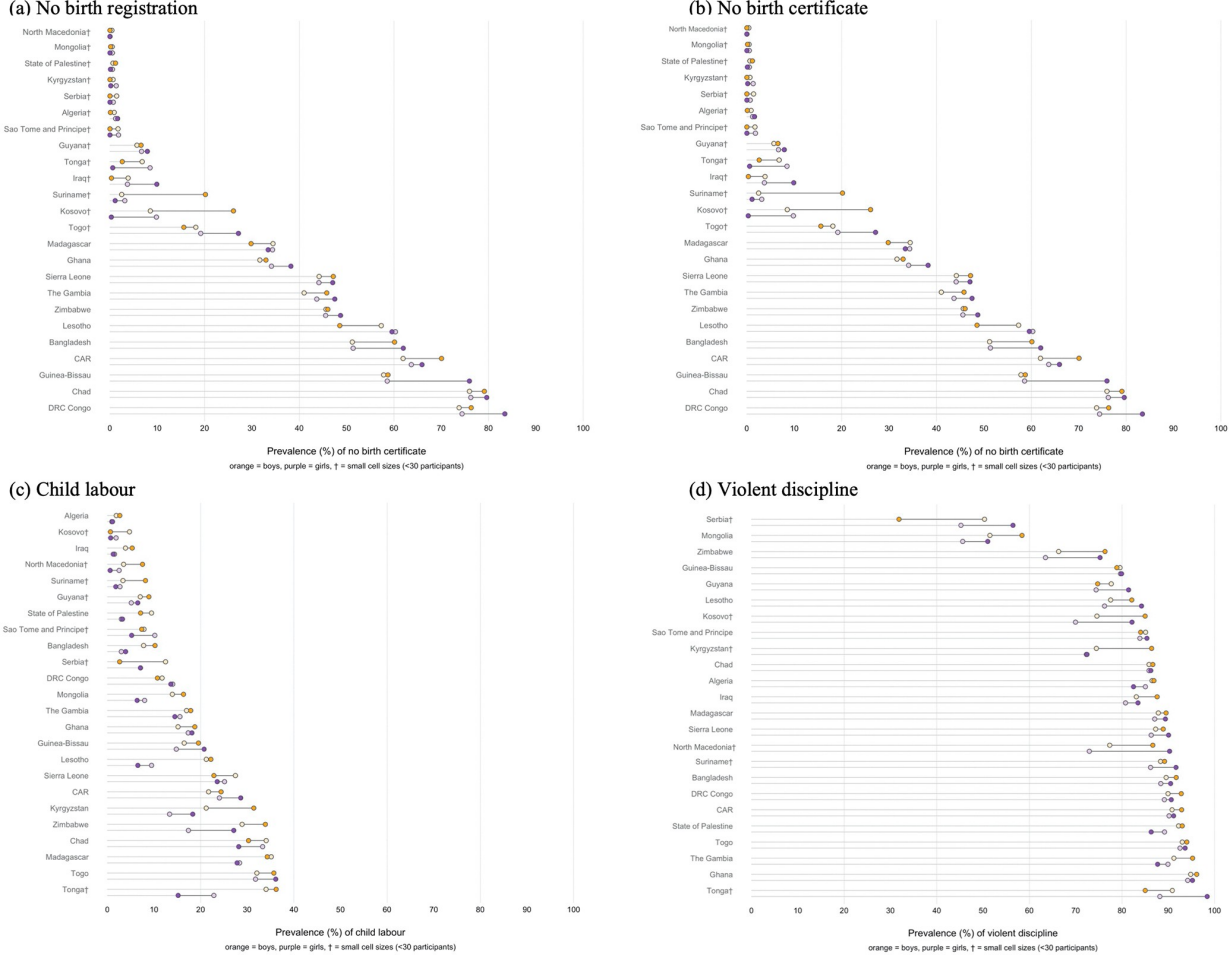
Prevalence gaps in birth registration and certification, child labour, and violent discipline by disability. Figure shows country specific age adjusted prevalence differences among girls (purple) and boys (orange) with disabilities compared to girls and boys without disabilities (white). † indicates small cell sizes (<30 participants). Relative inequities are shown in Fig B in S1 Text.

The percentage of children with disabilities ranged from 4.0% in Serbia to 28% in CAR. In ten countries, more than 15% of children aged 2–17 years had a disability. Near-universal birth registration among children aged 2–4 years had been achieved by half of the countries (n = 12) where 32% or fewer children did not have their birth registered. Most (11/12) countries without universal birth registration were in Sub Saharan Africa: the percentage of children without birth registration ranged from 74.1% in Chad to 14.5% in Togo. The percentage of children aged 5–17 years engaged in child labour in the past week ranged from 2.0% in Algeria to 40% in Chad. The past-month prevalence of violent discipline among children aged 1–14 years ranged from 48% in Serbia to 95% in Ghana. Table 2 shows the percentage of children with disabilities, without birth registration, experiencing child labour and violent discipline disaggregated by disability in each country. Table A in S1 Text shows inequities in child protection outcomes by disability status in 24 countries. Fig 1 shows absolute inequities and Fig A in S1 Text shows relative inequities.

### Birth registration

Our analysis focuses on the countries that have not achieved near-universal birth registration (n = 12) or birth certificate coverage (n = 16) among children aged 2–4 years. Girls with disabilities were more likely to be unregistered compared to girls without disabilities in two countries—Guinea Bissau (aPR:, 1.37, 95% CI: 1.16, 1.62) and the Democratic Republic of Congo (aPR: 1.16, 95% CI: 1.00, 1.35) (Fig 1). In these countries, the prevalence gap in birth registration was 18.8%-points in Guinea Bissau and 9.7%-points in DRC. Among boys, we found relative inequities in birth registration by disability in one country, CAR (aPR: 1.15, 95% CI: 1.03, 1.29), where the prevalence gap was 7.9%-points. In addition, in three countries (The Gambia, CAR and Guinea-Bissau) prevalence gaps were 6.0–8.3% but aPRs were not significant.

Among the countries without complete birth certificate coverage, girls with disabilities were more likely to be without a birth certificate in Bangladesh (aPR: 1.20, 95% CI: 1.07, 1.36. prevalence gap: 10.6%-points) and Guinea Bissau (aPR: 1.29. 95% CI: 1.11, 1.51), where the prevalence gap was 17.4%-points. Among boys, there were inequities in no birth certification by disability in two countries: CAR (aPR: 1.12, 95% CI: 1.03, 1.22 and Bangladesh (aPR: 1.17, 95% CI: 1.08, 1.27) where prevalence gaps were 8–9%-points.

### Child labour

Among girls with disabilities aged 5–17 years, the prevalence of child labour in the past week was higher in two countries out of 24 countries examined—Guinea-Bissau (aPR: 1.42, 95% CI:1.13, 1.79) and Zimbabwe (aPR: 1.51, 95% CI: 1.17, 1.94) where 27.1% of girls with disabilities were engaged in child labour compared to 17.4% of girls without disabilities (prevalence gap of 9.7%-points). Among boys, we found inequities in child labour by disability in two countries (Bangladesh and Kyrgyzstan): the aPR and prevalence gap were largest in Kyrgyzstan: the prevalence of child labour was 31.4% among boys with disabilities compared to 21.2% (aPR: 1.44, 95% CI: 1.06, 1.96. Prevalence gap: 10.2%-points).

**Fig 2:**
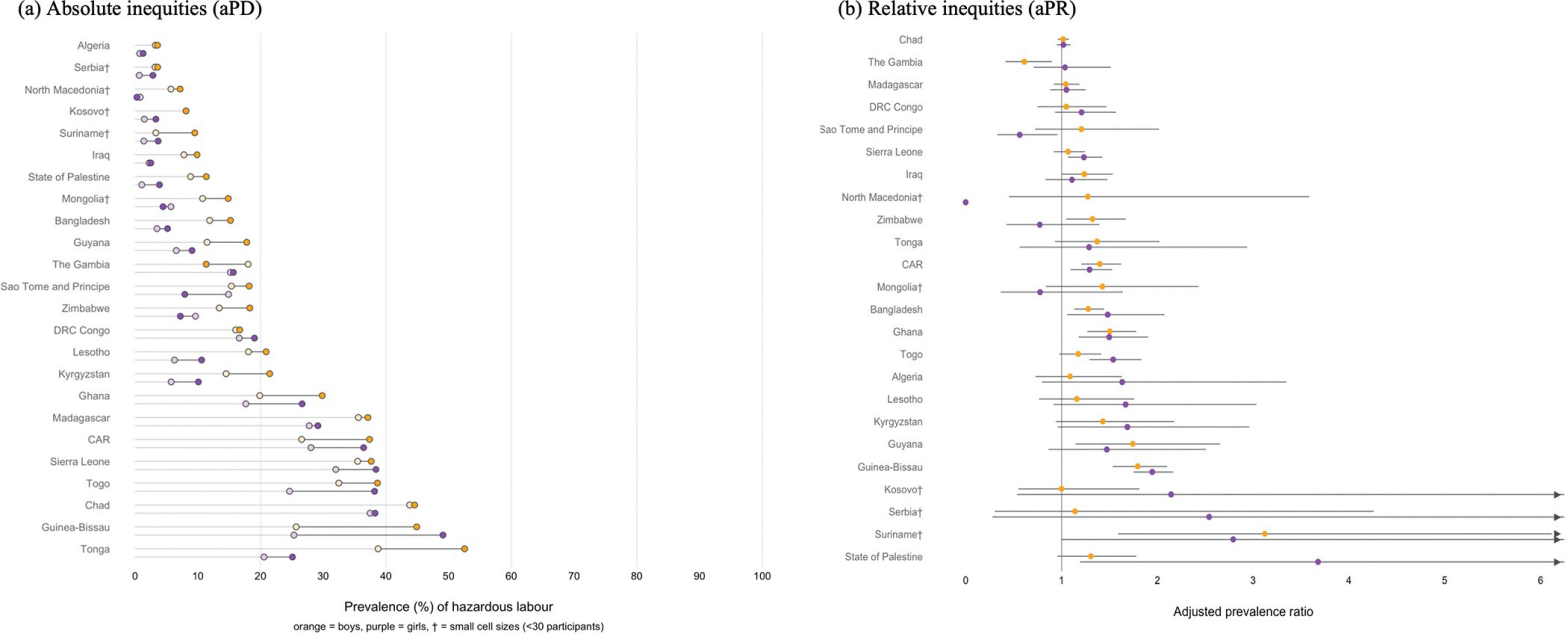
Absolute and relative inequities in hazardous labour by disability status. Figure shows (a) country specific age adjusted prevalence differences (aPDs) and (b) prevalence ratios (aPRs) among girls (purple) and boys (yellow). In panel (a), lightly shaded circles indicate the prevalence among children without disabilities. † indicates small overall cell sizes (<30 participants).

Hazardous work was higher among girls with disabilities in six countries: aPRs ranged from 1.23 in Sierra Leone (95% CI: 1.07, 1.42) to 1.95 in Guinea-Bissau (95% CI: 1.75, 2.16) (Fig 2). Although aPRs were larger in Palestine and Suriname, cell sizes were small. Among the six countries with relative inequities in hazardous labour by disability, prevalence gaps were largest among girls in Guinea Bissau (23.8%-points, hazardous labour was 49.1% among girls with disabilities vs. 25.3% among girls without disabilities) and Togo (13.6%-points; prevalence of 38.7% vs. 25.1%) and smallest in Bangladesh (1.7%-points, prevalence of 5.2% vs. 3.5%). Hazardous labour was higher among boys in seven countries and aPRs ranged from 1.24 in Iraq (95% CI: 1.00, 1.53) to 1.80 in Guinea-Bissau (95% CI: 1.54, 2.10). Prevalence gaps ranged from 2.1%-points in Iraq to 19.2%-points in Guinea-Bissau and were larger than 5%-points in four countries.

In a few countries, the prevalence of child labour was lower among boys and girls with disabilities. Among girls, in Chad (aPR: 0.86, 95% CI: 0.78, 0.95, prevalence difference 5.1%-points) and among boys, in Chad (aPR: 0.90, 95% CI: 0.83, 0.97) as well as Sierra Leone (aPR: 0.82, 95% CI: 0.72, 0.94) with prevalence differences less than 5%-points. Hazardous labour was only lower among boys in The Gambia (aPR: 0.61, 95% CI: 0.42, 0.89, prevalence difference: 6.7%-points).

### Violent discipline

Among girls, we found inequities in the prevalence of violent discipline in the past month by disability in four out of 24 countries (four additional countries had significant aPRs, however cell sizes were small): aPRs ranged from 1.02 (95% CI: 1.01, 1.04) in Bangladesh to 1.18 in Zimbabwe (95% CI: 1.10, 1.27), where the prevalence gap was 11.8%-points. In three countries, although aPRs were not significant, prevalence gaps larger than 5%-points (Mongolia, Serbia, Guyana). Among boys, there were inequities in violent discipline by disability in four countries (two additional countries had significant aPRs, however cell sizes were small). Zimbabwe had the largest aPR: the prevalence of violent discipline among boys with disabilities was 1.15 times (95% CI: 1.07, 1.23) higher than among boys without disability. Prevalence gaps were larger than 10%-points in Mongolia (6.9%-points) and Zimbabwe (10.0%-points).

**Fig 3:**
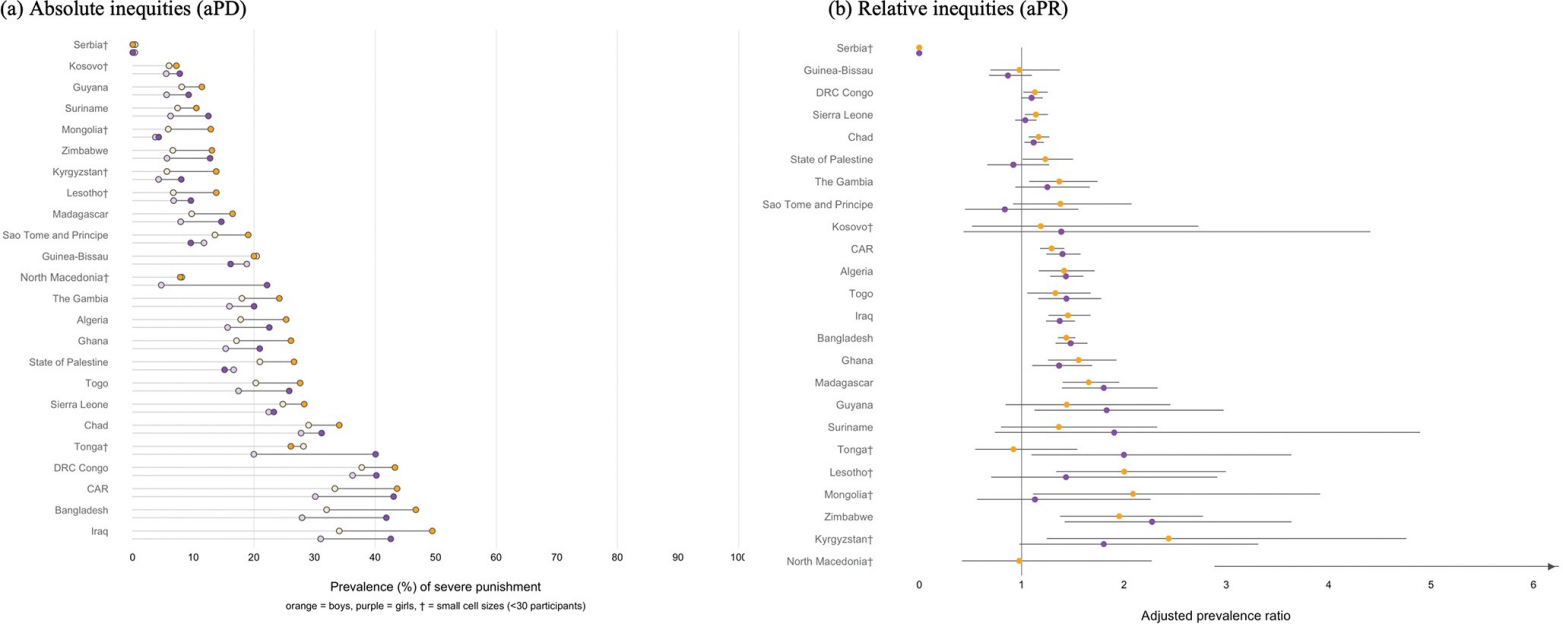
Absolute and relative inequities in severe punishment by disability status. Figure shows (a) country specific age adjusted prevalence differences (aPDs) and (b) prevalence ratios (aPRs) among girls (purple) and boys (yellow). In panel (a), lightly shaded circles indicate the prevalence among children without disabilities. † indicates small overall cell sizes (<30 participants).

Severe punishment was higher among girls with disabilities in nine countries: aPRs ranged from 1.12 in Chad (95% CI: 1.03, 1.21) to 2.27 in Zimbabwe (95% CI: 1.42, 3.63) (Fig 3). Among countries with significant aPRs, prevalence differences were larger than 5%-points in eight countries, larger than 10%-points in three countries, and as large as 13.9%-points in Bangladesh. Among boys, there were significant inequities in severe punishment by disability in 13 countries: aPRs ranged from 1.13 in DRC Congo (95% CI: 1.02, 1.25) to 1.95 in Zimbabwe (95% CI: 1.38, 2.77). In three countries, the prevalence of severe punishment among boys with disabilities was at least 1.5 times higher than among boys without disabilities. Among countries with significant aPRs, prevalence gaps were larger than 5%-points in 12 countries, and larger than 10%-points in 3 countries (CAR, Bangladesh and Iraq), and as large as 15.4%-points in Iraq.

There was no country where violent discipline or severe punishment were lower among girls or boys with disabilities, or where severe punishment was lower among boys or girls with disabilities.

## Discussion

We draw on nationally representative survey data from 323,436 children aged 2–17 years in 24 countries to estimate age-adjusted absolute and relative measures of inequity by disability status across six child protection outcomes. Our findings show that many countries have a high overall prevalence of non-registration, child labour, and violent discipline. For example, the prevalence of violent discipline was larger than 50% in 23 out of 24 countries and in 15 countries, caregivers reported that 4 out of 5 children had experienced a form of violent discipline in the past month. We find large variations in child protection outcomes by disability status, sex and by country. There were few countries with inequities in birth registration and child labour by disability among boys and girls. In contrast, many countries showed inequities in hazardous working conditions and severe punishment by disability. Importantly, we find few examples where countries have a lower prevalence of adverse child protection outcomes for boys and girls with disabilities compared to children without disabilities.

Evidence on birth registration and disability has been almost nonexistent [7]. We show that 12 countries have eliminated inequities in birth registration, demonstrating it is possible to achieve universal birth registration among children with and without disabilities. Given the importance of birth registration for school enrollment, age identification and access to social welfare schemes [27, 28] this is encouraging. However, we also show progress to ensure universal birth certification remains incomplete in most countries, and inequities for boys and girls with disabilities in access to birth certificates persist in several countries. These findings are consistent with a recent UNICEF analysis that pooled MICS data across countries and found that children with disabilities in urban areas and those in the poorest households were more likely to be unregistered than children without disabilities, and that the prevalence gap for children with disabilities became larger with age [7].

Our findings show large and prevalent inequities by disability in hazardous working conditions in six countries among girls and seven countries among boys out of 24 countries included, and often in different countries. These findings are consistent with two other studies using different samples of MICS data [7, 11], including a study drawing on data from 15 countries, which found that children with disabilities were 4% more likely than children without disabilities to be in child labour and 18% more likely to work under hazardous conditions [11].

A recent systematic review of global estimates on violence and disability found that emotional and physical violence were the most commonly reported forms of violence experienced by children and adolescents with disabilities [12]. Our findings extend and build on this evidence to show that inequities in violent discipline and severe punishment are different among girls and boys, both with regard to country context, direction, and magnitude of effect. We find that girls with disabilities were punished more using severe forms of violence in nine countries and boys were in 13 countries. Boys with disabilities experienced severe punishment more than boys without disabilities in more than half of the countries included, however the largest aPRs and prevalence gaps were among girls. Another global study reported that girls and young women with disabilities experience more violence than girls without disabilities [29], a UNICEF analysis pooled data across countries to also reveal a higher prevalence of violent discipline among children with disabilities [7] and a recent study using MICS data also found that children with disabilities were at higher risk of exposure to all forms of violent parental discipline in 17 LMICs [30]. However, these studies did not examine the risk among boys and girls separately.

This study is the first to describe multiple inequities concurrently for boys and girls with and without disabilities across a large number of countries. We use standardised measures to assess disability [31] and child protection outcomes. A potential limitation is that all outcomes were caregiver reported, which may lead to underreporting. Furthermore, our analyses are descriptive, and the use of cross-sectional data precludes our ability to comment on causality. The national level analysis we present could obscure sub-national inequities. Although MICS are among the largest population-level surveys on disability and violence in LMICs, cell sizes for some indicators cell sizes were below 30 children. It was therefore not possible to disaggregate by disability type or by other sociodemographic variables (e.g., wealth, rurality). MICS are household surveys and do not include children who are street connected, incarcerated, or living in residential care and our results should not be generalised to these groups.

In accordance with the SDG’s principle to “leave no one behind” [4], our findings underscore the importance of examining inequities in child protection outcomes using an intersectional lens, and of estimating inequities. We find that average estimates, as well as estimates disaggregated by disability, can mask gender differences in child protection outcomes and increase the invisibility of violence for girls and boys with disabilities [9]. Equity-oriented monitoring of child rights by disability status and by disaggregating data by disability and sex [9] allows governments, policymakers and researchers to explicitly to develop, and monitor, targets based on a reduction in inequities [32]. This will be particularly important to ensure efforts continue to reduce non-registration, child labour and violent discipline do not widen or create gaps for children with disabilities [21, 24, 33]. The choice of inequality measure is particularly important in evaluating whether—and how much—inequities are changing over time and we find that both relative and absolute inequities should be explored and reported [34].

We highlight the importance of ensuring efforts to prevent non-registration, child labour, and violent discipline are responsive to children with disabilities. Interventions, programs, and policies to prevent violence and exploitation among children will only be successful if they do not widen inequities between children with and without disabilities. Given that current evidence suggests that that interventions often ignore children with disabilities, or are not effective among children with disabilities, there is also a need to consider intersectionality in the design and evaluation of interventions to make sure violence prevention programming is responsive to children with disabilities [35]. These data and this evidence are important to guide prevention and to hold governments and policy makers accountable for preventing violence, protecting child rights, and improving the health and well-being of children with disabilities.

## Supporting information

S1 Text (PDF)
