# Supplementary material for: Inequities in birth registration, violent discipline, and child labour by disability status and sex: Evidence from the Multiple Indicator Cluster Surveys in 24 countries
Source: PLOS Glob Public Health. 2023 May 24;3(5):e0001827. doi: 10.1371/journal.pgph.0001827 (PMC10208457; doi:10.1371/journal.pgph.0001827)
Supplement: S1 Text — (PDF) [file pgph.0001827.s001.pdf]

SUPPLEMENTARTY MATERIAL

Inequities in birth registration, violent discipline, and child labour by disability status and sex: Evidence from the Multiple Indicator Cluster Surveys in 24 countries

Tables

Table A: Inequities in child protection outcomes by disability status in 24 countries

Figures

Fig A: Relative inequities in birth registration, birth certification, child labour, and violent discipline by disability status

Table A: Inequities in child protection outcomes by disability status in 24 countries

A. Birth registration						
			No birth registration among girls			
Country	All children n/N (%)	Girls n/N (%)	No disability n/N (%)	With disability n/N (%)	Age-adj percentage ratio (95% CI)	Age-adj percentage difference
**EAP**						
Mongolia	1/3,796 (0)	0/1,822 (0)	0/1,794 (0)	0/28 (0)	--	-0.00
Tonga	14/859 (2)	6/386 (2)	6/366 (2)	0/20 (1)	-	-0.95
**ECA**						
Kosovo	10/923 (1)	3/427 (1)	3/419 (1)	0/8 (0)	-	-0.69
Kyrgyzstan	9/2,167 (0)	7/1,069 (1)	7/1,060 (1)	0/9 (0)	-	-0.62
North Macedonia	0/936 (0)	0/437 (0)	0/429 (0)	0/8 (0)	1.00 (0.47 to 2.14)	-0.00
Serbia	1/1,163 (0)	0/583 (0)	0/573 (0)	0/10 (0)	1.00 (0.50 to 2.01)	0.00
**LAC**						
Guyana	23/1,692 (1)	14/813 (2)	13/796 (2)	1/17 (7)	5.06 (0.98 to 26.14)	5.01
Suriname	20/2,697 (1)	10/1,284 (1)	10/1,245 (1)	0/39 (0)	-	-1.03
**MENA**						
Algeria	37/9,070 (0)	24/4,414 (1)	23/4,336 (1)	1/78 (1)	3.64 (0.45 to 29.27)	0.82
Iraq	56/10,162 (1)	26/4,971 (1)	26/4,855 (1)	0/116 (0)	-	-0.89
State of Palestine	13/3,704 (0)	7/1,782 (0)	7/1,748 (0)	0/34 (0)	-	-0.31
**SA**						
Bangladesh	5,173/14,048 (37)	2,476/6,755 (37)	2,418/6,610 (37)	58/145 (42)	1.15 (0.91 to 1.44)	5.40
**SSA**						
CAR	2,786/5,430 (51)	1,435/2,737 (52)	1,231/2,379 (52)	204/358 (54)	1.04 (0.90 to 1.20)	2.29
Chad	10,476/14,141 (74)	5,165/6,965 (74)	4,670/6,304 (74)	495/661 (75)	1.01 (0.95 to 1.06)	0.46
DRC Congo	8,877/12,745 (70)	4,524/6,456 (70)	4,261/6,090 (70)	263/366 (80)	1.16 (1.00 to 1.35)	9.73
Ghana	1,381/5,349 (26)	729/2,715 (27)	652/2,483 (26)	77/232 (34)	1.31 (0.95 to 1.80)	7.95

No birth registration among girls

Country	All children n/N (%)	Girls n/N (%)	No disability n/N (%)	With disability n/N (%)	Age-adj percentage ratio (95% CI)	Age-adj percentage difference
Guinea-Bissau	2,218/4,602 (48)	1,099/2,239 (49)	1,049/2,156 (49)	50/83 (67)	1.37 (1.16 to 1.62)	18.78
Lesotho	1,053/2,008 (52)	553/1,028 (54)	523/967 (54)	30/61 (53)	0.98 (0.70 to 1.36)	-1.20
Madagascar	1,372/7,021 (20)	678/3,450 (20)	626/3,171 (20)	52/279 (20)	1.03 (0.75 to 1.43)	0.58
Sao Tome and Principe	14/1,212 (1)	7/586 (1)	7/556 (1)	0/30 (0)	-	-1.59
Sierra Leone	1,215/7,077 (17)	640/3,569 (18)	609/3,366 (18)	31/203 (15)	0.84 (0.61 to 1.17)	-2.74
The Gambia	2,073/6,137 (34)	1,058/2,996 (35)	991/2,839 (35)	67/157 (41)	1.16 (0.93 to 1.46)	5.81
Togo	433/2,980 (15)	218/1,441 (15)	200/1,361 (15)	18/80 (21)	1.37 (0.74 to 2.54)	6.04
Zimbabwe	1,592/3,747 (42)	809/1,905 (42)	783/1,848 (42)	26/57 (44)	1.03 (0.78 to 1.36)	1.22

No birth registration among boys

Country	All children n/N (%)	Boys n/N (%)	No disability n/N (%)	With disability n/N (%)	Age-adj percentage ratio (95% CI)	Age-adj percentage difference
**EAP**						
Mongolia	1/3,796 (0)	1/1,974 (0)	1/1,937 (0)	0/37 (0)	-	-0.01
Tonga	14/859 (2)	8/473 (2)	8/443 (2)	0/30 (0)	-	-2.41
**ECA**						
Kosovo	10/923 (1)	7/496 (1)	6/481 (1)	1/15 (8)	6.77 (1.42 to 32.36)	6.87
Kyrgyzstan	9/2,167 (0)	2/1,098 (0)	2/1,077 (0)	0/21 (0)	-	-0.10
North Macedonia	0/936 (0)	0/499 (0)	0/492 (0)	0/7 (0)	1.00 (0.39 to 2.59)	0.00
Serbia	1/1,163 (0)	1/580 (0)	1/569 (0)	0/11 (0)	-	-0.15
**LAC**						
Guyana	23/1,692 (1)	9/879 (1)	8/843 (1)	1/36 (0)	0.38 (0.03 to 4.95)	-0.86
Suriname	20/2,697 (1)	10/1,413 (1)	7/1,373 (1)	3/40 (23)	31.12 (6.48 to 149.36)	22.01
**MENA**						
Algeria	37/9,070 (0)	13/4,656 (0)	13/4,525 (0)	0/131 (0)	-	-0.20

No birth registration among boys

Country	All children n/N (%)	Boys n/N (%)	No disability n/N (%)	With disability n/N (%)	Age-adj percentage ratio (95% CI)	Age-adj percentage difference
Iraq	56/10,162 (1)	30/5,191 (1)	30/5,019 (1)	0/172 (0)	-	-0.68
State of Palestine	13/3,704 (0)	6/1,922 (0)	5/1,876 (0)	1/46 (1)	5.80 (0.59 to 57.44)	0.90
**SA**						
Bangladesh	5,173/14,048 (37)	2,697/7,293 (37)	2,611/7,087 (37)	86/206 (42)	1.14 (0.95 to 1.36)	5.05
**SSA**						
CAR	2,786/5,430 (51)	1,351/2,693 (50)	1,159/2,359 (49)	192/334 (57)	1.15 (1.03 to 1.29)	7.92
Chad	10,476/14,141 (74)	5,311/7,176 (74)	4,780/6,477 (74)	531/699 (75)	1.02 (0.97 to 1.07)	1.39
DRC Congo	8,877/12,745 (70)	4,353/6,289 (69)	4,035/5,830 (69)	318/459 (73)	1.06 (0.93 to 1.22)	3.81
Ghana	1,381/5,349 (26)	652/2,634 (25)	579/2,364 (24)	73/270 (25)	1.01 (0.80 to 1.27)	0.16
Guinea-Bissau	2,218/4,602 (48)	1,119/2,363 (47)	1,064/2,262 (47)	55/101 (55)	1.17 (0.93 to 1.47)	8.27
Lesotho	1,053/2,008 (52)	500/980 (51)	454/890 (51)	46/90 (47)	0.92 (0.75 to 1.12)	-4.00
Madagascar	1,372/7,021 (20)	694/3,571 (19)	636/3,237 (20)	58/334 (15)	0.76 (0.54 to 1.06)	-4.32
Sao Tome and Principe	14/1,212 (1)	7/626 (1)	7/588 (1)	0/38 (0)	-	-1.18
Sierra Leone	1,215/7,077 (17)	575/3,508 (16)	524/3,261 (16)	51/247 (21)	1.34 (0.99 to 1.82)	5.24
The Gambia	2,073/6,137 (34)	1,015/3,141 (32)	928/2,913 (32)	87/228 (38)	1.19 (0.96 to 1.48)	5.95
Togo	433/2,980 (15)	215/1,539 (14)	198/1,417 (14)	17/122 (12)	0.89 (0.55 to 1.46)	-1.68
Zimbabwe	1,592/3,747 (42)	783/1,842 (43)	748/1,766 (42)	35/76 (43)	1.01 (0.75 to 1.37)	0.55

B. Birth certification

No birth certificate among girls

Country	All children n/N (%)	Girls n/N (%)	No disability n/N (%)	With disability n/N (%)	Age-adj percentage ratio (95% CI)	Age-adj percentage difference
**EAP**						
Mongolia	18/3,796 (0)	9/1,822 (0)	9/1,794 (1)	0/28 (0)	-	-0.74
Tonga	64/857 (7)	32/386 (8)	31/366 (8)	1/20 (1)	0.16 (0.03 to 0.94)	-7.89

No birth certificate among girls

Country	All children n/N (%)	Girls n/N (%)	No disability n/N (%)	With disability n/N (%)	Age-adj percentage ratio (95% CI)	Age-adj percentage difference
**ECA**						
Kosovo	85/921 (9)	41/426 (10)	41/418 (10)	0/8 (0)	-	-9.52
Kyrgyzstan	21/2,167 (1)	14/1,069 (1)	14/1,060 (1)	0/9 (0)	-	-1.15
North Macedonia	2/936 (0)	0/437 (0)	0/429 (0)	0/8 (0)	1.00 (0.47 to 2.14)	-0.00
Serbia	12/1,162 (1)	4/582 (1)	4/572 (1)	0/10 (0)	-	-1.11
**LAC**						
Guyana	105/1,691 (6)	55/812 (7)	53/795 (7)	2/17 (8)	1.23 (0.21 to 7.29)	1.23
Suriname	77/2,696 (3)	40/1,283 (3)	39/1,244 (3)	1/39 (1)	0.44 (0.06 to 3.38)	-2.04
**MENA**						
Algeria	94/9,070 (1)	54/4,414 (1)	52/4,336 (1)	2/78 (2)	1.34 (0.30 to 6.05)	0.42
Iraq	380/10,145 (4)	185/4,966 (4)	179/4,850 (4)	6/116 (10)	2.42 (0.89 to 6.63)	6.20
State of Palestine	22/3,704 (1)	9/1,782 (1)	9/1,748 (1)	0/34 (0)	-	-0.41
**SA**						
Bangladesh	7,229/14,041 (51)	3,479/6,753 (52)	3,395/6,608 (51)	84/145 (62)	1.20 (1.07 to 1.36)	10.61
**SSA**						
CAR	3,453/5,430 (64)	1,760/2,737 (64)	1,515/2,379 (64)	245/358 (66)	1.03 (0.94 to 1.14)	2.26
Chad	10,804/14,141 (76)	5,330/6,965 (77)	4,806/6,304 (76)	524/661 (80)	1.04 (0.99 to 1.10)	3.37
DRC Congo	9,469/12,745 (74)	4,823/6,456 (75)	4,531/6,090 (74)	292/366 (83)	1.14 (0.99 to 1.31)	9.04
Ghana	1,784/5,337 (33)	940/2,711 (35)	846/2,480 (34)	94/231 (38)	1.12 (0.83 to 1.51)	4.11
Guinea-Bissau	2,688/4,602 (58)	1,321/2,239 (59)	1,263/2,156 (59)	58/83 (76)	1.29 (1.11 to 1.51)	17.38
Lesotho	1,176/2,004 (59)	617/1,026 (60)	582/965 (60)	35/61 (60)	0.99 (0.75 to 1.29)	-0.73
Madagascar	2,397/7,021 (34)	1,179/3,450 (34)	1,089/3,171 (34)	90/279 (33)	0.97 (0.76 to 1.23)	-0.92
Sao Tome and Principe	20/1,212 (2)	10/586 (2)	10/556 (2)	0/30 (0)	-	-2.24
Sierra Leone	3,125/7,053 (44)	1,574/3,556 (44)	1,480/3,353 (44)	94/203 (47)	1.07 (0.91 to 1.25)	2.92

No birth certificate among girls

Country	All children n/N (%)	Girls n/N (%)	No disability n/N (%)	With disability n/N (%)	Age-adj percentage ratio (95% CI)	Age-adj percentage difference
The Gambia	2,620/6,132 (43)	1,319/2,993 (44)	1,239/2,836 (44)	80/157 (47)	1.09 (0.90 to 1.31)	3.81
Togo	562/2,980 (19)	284/1,441 (20)	261/1,361 (19)	23/80 (27)	1.38 (0.90 to 2.10)	7.96
Zimbabwe	1,716/3,747 (46)	872/1,905 (46)	842/1,848 (46)	30/57 (49)	1.07 (0.85 to 1.34)	3.15

No birth certificate among boys

Country	All children n/N (%)	Boys n/N (%)	No disability n/N (%)	With disability n/N (%)	Age-adj percentage ratio (95% CI)	Age-adj percentage difference
**EAP**						
Mongolia	18/3,796 (0)	9/1,974 (0)	9/1,937 (0)	0/37 (0)	-	-0.32
Tonga	64/857 (7)	32/471 (7)	30/441 (7)	2/30 (3)	0.41 (0.08 to 2.18)	-4.21
**ECA**						
Kosovo	85/921 (9)	44/495 (9)	41/481 (9)	3/14 (26)	3.10 (1.40 to 6.85)	17.57
Kyrgyzstan	21/2,167 (1)	7/1,098 (1)	7/1,077 (1)	0/21 (0)	-	-0.66
North Macedonia	2/936 (0)	2/499 (0)	2/492 (0)	0/7 (0)	-	-0.60
Serbia	12/1,162 (1)	8/580 (1)	8/569 (1)	0/11 (0)	-	-1.94
**LAC**						
Guyana	105/1,691 (6)	50/879 (6)	48/843 (6)	2/36 (7)	1.12 (0.24 to 5.27)	0.87
Suriname	77/2,696 (3)	37/1,413 (3)	34/1,373 (2)	3/40 (20)	7.28 (1.55 to 34.25)	17.70
**MENA**						
Algeria	94/9,070 (1)	40/4,656 (1)	40/4,525 (1)	0/131 (0)	-	-0.79
Iraq	380/10,145 (4)	195/5,179 (4)	192/5,007 (4)	3/172 (0)	0.22 (0.05 to 0.98)	-3.52
State of Palestine	22/3,704 (1)	13/1,922 (1)	12/1,876 (1)	1/46 (1)	1.95 (0.21 to 18.09)	0.53
**SA**						
Bangladesh	7,229/14,041 (51)	3,750/7,288 (51)	3,626/7,082 (51)	124/206 (60)	1.17 (1.08 to 1.27)	8.93
**SSA**						

No birth certificate among boys

Country	All children n/N (%)	Boys n/N (%)	No disability n/N (%)	With disability n/N (%)	Age-adj percentage ratio (95% CI)	Age-adj percentage difference
CAR	3,453/5,430 (64)	1,693/2,693 (63)	1,461/2,359 (62)	232/334 (70)	1.12 (1.03 to 1.22)	8.13
Chad	10,804/14,141 (76)	5,474/7,176 (76)	4,920/6,477 (76)	554/699 (79)	1.04 (0.99 to 1.10)	3.15
DRC Congo	9,469/12,745 (74)	4,646/6,289 (74)	4,301/5,830 (74)	345/459 (76)	1.04 (0.91 to 1.18)	2.55
Ghana	1,784/5,337 (33)	844/2,626 (32)	746/2,356 (32)	98/270 (33)	1.04 (0.86 to 1.26)	1.29
Guinea-Bissau	2,688/4,602 (58)	1,367/2,363 (58)	1,308/2,262 (58)	59/101 (59)	1.02 (0.83 to 1.24)	0.91
Lesotho	1,176/2,004 (59)	559/978 (57)	509/888 (57)	50/90 (49)	0.84 (0.71 to 1.01)	-8.76
Madagascar	2,397/7,021 (34)	1,218/3,571 (34)	1,115/3,237 (34)	103/334 (30)	0.85 (0.69 to 1.04)	-4.63
Sao Tome and Principe	20/1,212 (2)	10/626 (2)	10/588 (2)	0/38 (0)	-	-1.77
Sierra Leone	3,125/7,053 (44)	1,551/3,497 (44)	1,436/3,250 (44)	115/247 (47)	1.07 (0.87 to 1.31)	3.00
The Gambia	2,620/6,132 (43)	1,301/3,139 (41)	1,194/2,911 (41)	107/228 (46)	1.12 (0.94 to 1.33)	4.79
Togo	562/2,980 (19)	278/1,539 (18)	257/1,417 (18)	21/122 (16)	0.88 (0.56 to 1.38)	-2.53
Zimbabwe	1,716/3,747 (46)	844/1,842 (46)	806/1,766 (46)	38/76 (46)	1.01 (0.76 to 1.33)	0.39

C. Violent Discipline

Violent discipline among girls

Country	All children n/N (%)	Girls n/N (%)	No disability n/N (%)	With disability n/N (%)	Age-adj percentage ratio (95% CI)	Age-adj percentage difference
**EAP**						
Mongolia	4,866/9,941 (49)	2,201/4,804 (46)	2,098/4,601 (46)	103/203 (51)	1.12 (0.88 to 1.41)	5.40
Tonga	1,869/2,086 (90)	859/970 (89)	790/896 (88)	69/74 (98)	1.12 (1.04 to 1.21)	10.25
**ECA**						
Kosovo	1,916/2,626 (73)	859/1,218 (71)	801/1,145 (70)	58/73 (82)	1.17 (1.01 to 1.36)	12.20
Kyrgyzstan	3,946/5,356 (74)	1,876/2,594 (72)	1,783/2,466 (72)	93/128 (72)	1.00 (0.88 to 1.14)	0.14
North Macedonia	1,571/2,077 (76)	737/1,004 (73)	682/935 (73)	55/69 (90)	1.24 (1.06 to 1.45)	17.33
Serbia	1,229/2,573 (48)	564/1,245 (45)	549/1,214 (45)	15/31 (56)	1.25 (0.89 to 1.76)	11.23
**LAC**						

Violent discipline among girls

Country	All children n/N (%)	Girls n/N (%)	No disability n/N (%)	With disability n/N (%)	Age-adj percentage ratio (95% CI)	Age-adj percentage difference
Guyana	3,175/4,193 (76)	1,545/2,085 (74)	1,372/1,844 (74)	173/241 (81)	1.10 (0.96 to 1.25)	7.02
Suriname	5,052/5,771 (88)	2,450/2,831 (87)	2,229/2,587 (86)	221/244 (92)	1.06 (1.00 to 1.13)	5.52
**MENA**						
Algeria	18,846/22,021 (86)	8,979/10,599 (85)	7,991/9,396 (85)	988/1,203 (82)	0.97 (0.94 to 1.01)	-2.57
Iraq	18,513/22,493 (82)	8,852/10,926 (81)	7,804/9,664 (81)	1,048/1,262 (83)	1.03 (0.99 to 1.07)	2.69
State of Palestine	7,117/7,869 (90)	3,372/3,809 (89)	3,140/3,521 (89)	232/288 (86)	0.97 (0.92 to 1.02)	-2.88
**SA**						
Bangladesh	39,542/44,420 (89)	19,228/21,739 (88)	18,103/20,486 (88)	1,125/1,253 (90)	1.02 (1.01 to 1.04)	2.13
**SSA**						
CAR	9,604/10,564 (91)	4,778/5,276 (91)	3,694/4,097 (90)	1,084/1,179 (91)	1.01 (0.98 to 1.04)	1.00
Chad	22,670/26,423 (86)	11,270/13,144 (86)	9,084/10,583 (86)	2,186/2,561 (86)	1.00 (0.98 to 1.03)	0.38
DRC Congo	21,943/24,448 (90)	11,014/12,338 (89)	9,442/10,592 (89)	1,572/1,746 (91)	1.02 (0.99 to 1.04)	1.49
Ghana	12,044/12,720 (95)	5,987/6,348 (94)	5,079/5,391 (94)	908/957 (95)	1.01 (0.98 to 1.04)	1.02
Guinea-Bissau	7,558/9,454 (80)	3,769/4,718 (80)	3,404/4,272 (80)	365/446 (80)	1.00 (0.94 to 1.07)	0.22
Lesotho	4,564/5,905 (77)	2,288/2,984 (77)	2,119/2,780 (76)	169/204 (84)	1.10 (1.03 to 1.19)	7.99
Madagascar	14,525/16,609 (87)	7,164/8,232 (87)	6,355/7,300 (87)	809/932 (89)	1.03 (0.99 to 1.07)	2.31
Sao Tome and Principe	2,468/2,921 (84)	1,198/1,428 (84)	1,023/1,220 (84)	175/208 (85)	1.02 (0.96 to 1.08)	1.52
Sierra Leone	14,078/16,146 (87)	7,046/8,120 (87)	5,885/6,819 (86)	1,161/1,301 (90)	1.04 (1.02 to 1.07)	3.75
The Gambia	9,875/10,909 (91)	5,048/5,620 (90)	4,690/5,216 (90)	358/404 (88)	0.98 (0.92 to 1.03)	-2.23
Togo	6,529/7,029 (93)	3,221/3,479 (93)	2,771/2,994 (93)	450/485 (94)	1.01 (0.98 to 1.04)	1.10
Zimbabwe	6,202/9,467 (66)	3,011/4,698 (64)	2,798/4,408 (63)	213/290 (75)	1.18 (1.10 to 1.27)	11.78

Violent discipline among boys

Country	All children n/N (%)	Boys n/N (%)	No disability n/N (%)	With disability n/N (%)	Age-adj percentage ratio (95% CI)	Age-adj percentage difference
**EAP**						
Mongolia	4,866/9,941 (49)	2,665/5,137 (52)	2,526/4,907 (51)	139/230 (58)	1.13 (0.96 to 1.31)	6.93
Tonga	1,869/2,086 (90)	1,010/1,116 (91)	926/1,019 (91)	84/97 (85)	0.94 (0.82 to 1.07)	-5.86
**ECA**						
Kosovo	1,916/2,626 (73)	1,057/1,408 (75)	983/1,318 (75)	74/90 (85)	1.14 (1.02 to 1.27)	10.41
Kyrgyzstan	3,946/5,356 (74)	2,070/2,762 (75)	1,942/2,608 (74)	128/154 (86)	1.16 (1.06 to 1.26)	11.93
North Macedonia	1,571/2,077 (76)	834/1,073 (78)	775/1,002 (77)	59/71 (87)	1.12 (0.98 to 1.27)	9.30
Serbia	1,229/2,573 (48)	665/1,328 (50)	644/1,280 (50)	21/48 (32)	0.63 (0.28 to 1.40)	-18.47
**LAC**						
Guyana	3,175/4,193 (76)	1,630/2,108 (77)	1,430/1,841 (78)	200/267 (75)	0.96 (0.86 to 1.07)	-2.93
Suriname	5,052/5,771 (88)	2,602/2,940 (89)	2,344/2,654 (88)	258/286 (89)	1.01 (0.96 to 1.07)	0.87
**MENA**						
Algeria	18,846/22,021 (86)	9,867/11,422 (86)	8,423/9,739 (86)	1,444/1,683 (87)	1.00 (0.98 to 1.03)	0.41
Iraq	18,513/22,493 (82)	9,661/11,567 (84)	8,161/9,822 (83)	1,500/1,745 (88)	1.05 (0.99 to 1.12)	4.49
State of Palestine	7,117/7,869 (90)	3,745/4,060 (92)	3,355/3,637 (92)	390/423 (93)	1.01 (0.99 to 1.03)	0.79
**SA**						
Bangladesh	39,542/44,420 (89)	20,314/22,681 (90)	18,902/21,112 (90)	1,412/1,569 (92)	1.02 (1.01 to 1.04)	2.20
**SSA**						
CAR	9,604/10,564 (91)	4,826/5,288 (91)	3,756/4,136 (91)	1,070/1,152 (93)	1.02 (1.00 to 1.05)	2.09
Chad	22,670/26,423 (86)	11,400/13,279 (86)	9,107/10,610 (86)	2,293/2,669 (87)	1.01 (0.98 to 1.04)	0.82
DRC Congo	21,943/24,448 (90)	10,929/12,110 (90)	9,214/10,246 (90)	1,715/1,864 (93)	1.03 (1.01 to 1.06)	2.90
Ghana	12,044/12,720 (95)	6,057/6,372 (95)	5,035/5,308 (95)	1,022/1,064 (96)	1.01 (1.00 to 1.03)	1.30
Guinea-Bissau	7,558/9,454 (80)	3,789/4,736 (80)	3,428/4,308 (80)	361/428 (79)	0.99 (0.88 to 1.11)	-0.69
Lesotho	4,564/5,905 (77)	2,276/2,921 (78)	2,065/2,663 (78)	211/258 (82)	1.06 (0.96 to 1.16)	4.57

Violent discipline among boys

Country	All children n/N (%)	Boys n/N (%)	No disability n/N (%)	With disability n/N (%)	Age-adj percentage ratio (95% CI)	Age-adj percentage difference
Madagascar	14,525/16,609 (87)	7,361/8,377 (88)	6,423/7,312 (88)	938/1,065 (90)	1.02 (0.98 to 1.06)	1.67
Sao Tome and Principe	2,468/2,921 (84)	1,270/1,493 (85)	1,073/1,261 (85)	197/232 (84)	0.99 (0.92 to 1.06)	-1.07
Sierra Leone	14,078/16,146 (87)	7,032/8,026 (88)	5,760/6,601 (87)	1,272/1,425 (89)	1.02 (0.99 to 1.05)	1.65
The Gambia	9,875/10,909 (91)	4,827/5,289 (91)	4,402/4,826 (91)	425/463 (95)	1.04 (1.01 to 1.08)	4.05
Togo	6,529/7,029 (93)	3,308/3,550 (93)	2,806/3,016 (93)	502/534 (94)	1.01 (0.99 to 1.04)	0.99
Zimbabwe	6,202/9,467 (66)	3,191/4,769 (67)	2,920/4,403 (66)	271/366 (76)	1.15 (1.07 to 1.23)	10.01

D. Severe Punishment

Severe punishment among girls

Country	All children n/N (%)	Girls n/N (%)	No disability n/N (%)	With disability n/N (%)	Age-adj percentage ratio (95% CI)	Age-adj percentage difference
**EAP**						
Mongolia	501/9,976 (5)	187/4,825 (4)	173/4,622 (4)	14/203 (4)	1.13 (0.57 to 2.26)	0.54
Tonga	516/2,086 (25)	202/970 (21)	179/896 (20)	23/74 (40)	2.00 (1.10 to 3.63)	20.09
**ECA**						
Kosovo	154/2,623 (6)	68/1,214 (6)	63/1,141 (6)	5/73 (8)	1.39 (0.44 to 4.40)	2.23
Kyrgyzstan	285/5,356 (5)	117/2,594 (5)	105/2,466 (4)	12/128 (8)	1.80 (0.98 to 3.31)	3.74
North Macedonia	139/2,076 (7)	51/1,004 (5)	44/935 (5)	7/69 (22)	6.60 (2.89 to 15.09)	17.45
Serbia	9/2,574 (0)	4/1,246 (0)	4/1,215 (0)	0/31 (0)	-	-0.36
**LAC**						
Guyana	310/4,198 (7)	129/2,085 (6)	103/1,845 (6)	26/240 (9)	1.83 (1.13 to 2.97)	3.63
Suriname	416/5,762 (7)	190/2,825 (7)	161/2,583 (6)	29/242 (12)	1.90 (0.74 to 4.89)	6.25
**MENA**						
Algeria	3,828/22,011 (17)	1,721/10,597 (16)	1,469/9,390 (16)	252/1,207 (23)	1.43 (1.28 to 1.60)	6.88
Iraq	7,600/22,484 (34)	3,496/10,921 (32)	2,995/9,658 (31)	501/1,263 (43)	1.37 (1.24 to 1.52)	11.56

Severe punishment among girls

Country	All children n/N (%)	Girls n/N (%)	No disability n/N (%)	With disability n/N (%)	Age-adj percentage ratio (95% CI)	Age-adj percentage difference
State of Palestine	1,506/7,856 (19)	634/3,801 (17)	585/3,514 (17)	49/287 (15)	0.92 (0.67 to 1.27)	-1.50
**SA**						
Bangladesh	13,597/44,418 (31)	6,174/21,739 (28)	5,723/20,486 (28)	451/1,253 (42)	1.48 (1.33 to 1.64)	13.90
**SSA**						
CAR	3,607/10,554 (34)	1,728/5,270 (33)	1,234/4,096 (30)	494/1,174 (43)	1.40 (1.24 to 1.57)	12.92
Chad	7,785/26,464 (29)	3,752/13,162 (29)	2,942/10,593 (28)	810/2,569 (31)	1.12 (1.03 to 1.21)	3.40
DRC Congo	9,308/24,423 (38)	4,574/12,329 (37)	3,840/10,586 (36)	734/1,743 (40)	1.10 (1.00 to 1.20)	3.93
Ghana	2,240/12,721 (18)	1,047/6,348 (16)	827/5,391 (15)	220/957 (21)	1.37 (1.11 to 1.69)	5.61
Guinea-Bissau	1,900/9,454 (20)	899/4,718 (19)	804/4,272 (19)	95/446 (16)	0.87 (0.68 to 1.10)	-2.67
Lesotho	413/5,896 (7)	206/2,977 (7)	187/2,773 (7)	19/204 (10)	1.43 (0.71 to 2.91)	2.85
Madagascar	1,595/16,609 (10)	705/8,232 (9)	577/7,300 (8)	128/932 (15)	1.80 (1.40 to 2.32)	6.71
Sao Tome and Principe	380/2,917 (13)	168/1,424 (12)	143/1,217 (12)	25/207 (10)	0.84 (0.45 to 1.55)	-2.18
Sierra Leone	3,973/16,126 (25)	1,889/8,110 (23)	1,528/6,810 (22)	361/1,300 (23)	1.04 (0.94 to 1.14)	0.84
The Gambia	1,928/10,898 (18)	937/5,611 (17)	831/5,208 (16)	106/403 (20)	1.25 (0.94 to 1.66)	4.07
Togo	1,414/7,027 (20)	648/3,475 (19)	522/2,990 (17)	126/485 (26)	1.44 (1.17 to 1.77)	8.36
Zimbabwe	622/9,468 (7)	283/4,698 (6)	249/4,408 (6)	34/290 (13)	2.27 (1.42 to 3.63)	7.11

Severe punishment among boys

Country	All children n/N (%)	Boys n/N (%)	No disability n/N (%)	With disability n/N (%)	Age-adj percentage ratio (95% CI)	Age-adj percentage difference
**EAP**						
Mongolia	501/9,976 (5)	314/5,151 (6)	288/4,919 (6)	26/232 (13)	2.09 (1.11 to 3.91)	7.02
Tonga	516/2,086 (25)	314/1,116 (28)	287/1,019 (28)	27/97 (26)	0.92 (0.55 to 1.54)	-2.09
**ECA**						
Kosovo	154/2,623 (6)	86/1,409 (6)	79/1,319 (6)	7/90 (7)	1.19 (0.52 to 2.72)	1.21

Severe punishment among boys

Country	All children n/N (%)	Boys n/N (%)	No disability n/N (%)	With disability n/N (%)	Age-adj percentage ratio (95% CI)	Age-adj percentage difference
Kyrgyzstan	285/5,356 (5)	168/2,762 (6)	147/2,608 (6)	21/154 (14)	2.44 (1.25 to 4.76)	8.14
North Macedonia	139/2,076 (7)	88/1,072 (8)	81/1,001 (8)	7/71 (8)	0.98 (0.42 to 2.27)	-0.25
Serbia	9/2,574 (0)	5/1,328 (0)	5/1,280 (0)	0/48 (0)	-	-0.65
**LAC**						
Guyana	310/4,198 (7)	181/2,113 (9)	149/1,845 (8)	32/268 (11)	1.44 (0.85 to 2.45)	3.33
Suriname	416/5,762 (7)	226/2,937 (8)	196/2,651 (7)	30/286 (10)	1.36 (0.80 to 2.32)	3.10
**MENA**						
Algeria	3,828/22,011 (17)	2,107/11,414 (18)	1,732/9,729 (18)	375/1,685 (25)	1.42 (1.17 to 1.71)	7.51
Iraq	7,600/22,484 (34)	4,104/11,563 (35)	3,345/9,817 (34)	759/1,746 (49)	1.45 (1.26 to 1.67)	15.36
State of Palestine	1,506/7,856 (19)	872/4,055 (22)	762/3,633 (21)	110/422 (27)	1.23 (1.01 to 1.50)	5.64
**SA**						
Bangladesh	13,597/44,418 (31)	7,423/22,679 (33)	6,753/21,111 (32)	670/1,568 (47)	1.44 (1.36 to 1.52)	14.73
**SSA**						
CAR	3,607/10,554 (34)	1,879/5,284 (36)	1,378/4,132 (33)	501/1,152 (44)	1.29 (1.18 to 1.41)	10.27
Chad	7,785/26,464 (29)	4,033/13,302 (30)	3,083/10,626 (29)	950/2,676 (34)	1.17 (1.07 to 1.27)	5.06
DRC Congo	9,308/24,423 (38)	4,734/12,094 (39)	3,866/10,231 (38)	868/1,863 (43)	1.13 (1.02 to 1.25)	5.47
Ghana	2,240/12,721 (18)	1,193/6,373 (19)	909/5,308 (17)	284/1,065 (26)	1.56 (1.26 to 1.92)	8.97
Guinea-Bissau	1,900/9,454 (20)	1,001/4,736 (21)	881/4,308 (20)	120/428 (20)	0.98 (0.70 to 1.37)	-0.45
Lesotho	413/5,896 (7)	207/2,919 (7)	178/2,660 (7)	29/259 (14)	2.00 (1.34 to 2.99)	7.09
Madagascar	1,595/16,609 (10)	890/8,377 (11)	711/7,312 (10)	179/1,065 (16)	1.66 (1.40 to 1.95)	6.75
Sao Tome and Principe	380/2,917 (13)	212/1,493 (14)	171/1,261 (14)	41/232 (19)	1.38 (0.92 to 2.07)	5.49
Sierra Leone	3,973/16,126 (25)	2,084/8,016 (26)	1,633/6,594 (25)	451/1,422 (28)	1.14 (1.03 to 1.25)	3.53
The Gambia	1,928/10,898 (18)	991/5,287 (19)	869/4,824 (18)	122/463 (24)	1.37 (1.08 to 1.74)	6.18
Togo	1,414/7,027 (20)	766/3,552 (22)	613/3,018 (20)	153/534 (28)	1.33 (1.06 to 1.67)	7.31

Severe punishment among boys

Country	All children n/N (%)	Boys n/N (%)	No disability n/N (%)	With disability n/N (%)	Age-adj percentage ratio (95% CI)	Age-adj percentage difference
Zimbabwe	622/9,468 (7)	339/4,770 (7)	291/4,404 (7)	48/366 (13)	1.95 (1.38 to 2.77)	6.46

E. Child labour

Child labour among girls

Country	All children n/N (%)	Girls n/N (%)	No disability n/N (%)	With disability n/N (%)	Age-adj percentage ratio (95% CI)	Age-adj percentage difference
**EAP**						
Mongolia	817/7,314 (11)	286/3,538 (8)	265/3,321 (8)	21/217 (6)	0.79 (0.37 to 1.68)	-1.61
Tonga	456/1,623 (28)	171/776 (22)	159/696 (23)	12/80 (15)	0.60 (0.29 to 1.22)	-7.68
**ECA**						
Kosovo	77/2,345 (3)	19/1,060 (2)	18/968 (2)	1/92 (1)	0.47 (0.06 to 3.93)	-1.17
Kyrgyzstan	695/3,889 (18)	246/1,835 (13)	226/1,695 (13)	20/140 (18)	1.32 (0.86 to 2.03)	4.96
North Macedonia	46/1,428 (3)	17/708 (2)	16/637 (3)	1/71 (1)	0.06 (0.01 to 0.41)	-1.93
Serbia	168/1,726 (10)	58/815 (7)	56/790 (7)	2/25 (7)	1.00 (0.32 to 3.17)	-0.00
**LAC**						
Guyana	209/3,182 (7)	93/1,626 (6)	69/1,348 (5)	24/278 (7)	1.29 (0.57 to 2.92)	1.39
Suriname	121/3,867 (3)	48/1,923 (2)	45/1,672 (3)	3/251 (2)	0.64 (0.20 to 2.11)	-0.90
**MENA**						
Algeria	235/16,243 (1)	70/7,731 (1)	60/6,308 (1)	10/1,423 (1)	1.11 (0.44 to 2.84)	0.15
Iraq	449/15,486 (3)	109/7,411 (1)	91/5,994 (2)	18/1,417 (1)	0.86 (0.44 to 1.68)	-0.27
State of Palestine	323/5,251 (6)	77/2,512 (3)	66/2,205 (3)	11/307 (3)	1.07 (0.49 to 2.34)	0.24
**SA**						
Bangladesh	2,178/39,265 (6)	585/19,060 (3)	529/17,746 (3)	56/1,314 (4)	1.28 (0.78 to 2.11)	0.92
**SSA**						
CAR	1,431/5,972 (24)	756/2,970 (25)	488/2,031 (24)	268/939 (29)	1.18 (0.97 to 1.43)	4.57

Child labour among girls

Country	All children n/N (%)	Girls n/N (%)	No disability n/N (%)	With disability n/N (%)	Age-adj percentage ratio (95% CI)	Age-adj percentage difference
Chad	4,722/14,608 (32)	2,358/7,401 (32)	1,714/5,148 (33)	644/2,253 (28)	0.86 (0.78 to 0.95)	-5.12
DRC Congo	1,843/13,941 (13)	1,017/7,040 (14)	765/5,463 (14)	252/1,577 (14)	0.97 (0.73 to 1.29)	-0.40
Ghana	1,469/8,927 (16)	753/4,376 (17)	607/3,504 (17)	146/872 (18)	1.05 (0.85 to 1.29)	0.80
Guinea-Bissau	959/5,835 (16)	463/2,985 (16)	379/2,564 (15)	84/421 (21)	1.42 (1.13 to 1.79)	5.96
Lesotho	755/4,918 (15)	229/2,439 (9)	213/2,251 (9)	16/188 (7)	0.69 (0.32 to 1.45)	-2.94
Madagascar	3,745/11,915 (31)	1,676/5,966 (28)	1,460/5,149 (28)	216/817 (28)	0.98 (0.85 to 1.14)	-0.53
Sao Tome and Principe	182/2,165 (8)	99/1,093 (9)	87/856 (10)	12/237 (5)	0.51 (0.25 to 1.05)	-4.96
Sierra Leone	2,776/10,863 (26)	1,358/5,530 (25)	1,071/4,262 (25)	287/1,268 (24)	0.93 (0.80 to 1.09)	-1.57
The Gambia	924/5,649 (16)	490/3,111 (16)	441/2,836 (16)	49/275 (14)	0.92 (0.60 to 1.40)	-1.09
Togo	1,594/4,917 (32)	793/2,448 (32)	618/1,945 (32)	175/503 (36)	1.13 (0.95 to 1.35)	4.33
Zimbabwe	1,672/7,005 (24)	610/3,383 (18)	538/3,098 (17)	72/285 (27)	1.51 (1.17 to 1.94)	9.74

Child labour among boys

Country	All children n/N (%)	Girls n/N (%)	No disability n/N (%)	With disability n/N (%)	Age-adj percentage ratio (95% CI)	Age-adj percentage difference
**EAP**						
Mongolia	817/7,314 (11)	531/3,776 (14)	495/3,554 (14)	36/222 (16)	1.20 (0.73 to 1.97)	2.39
Tonga	456/1,623 (28)	285/847 (34)	259/761 (34)	26/86 (36)	1.07 (0.72 to 1.58)	2.17
**ECA**						
Kosovo	77/2,345 (3)	58/1,285 (5)	56/1,184 (5)	2/101 (1)	0.29 (0.07 to 1.20)	-4.08
Kyrgyzstan	695/3,889 (18)	449/2,054 (22)	402/1,897 (21)	47/157 (31)	1.44 (1.06 to 1.96)	10.21
North Macedonia	46/1,428 (3)	29/720 (4)	22/634 (3)	7/86 (8)	2.33 (0.71 to 7.63)	4.06
Serbia	168/1,726 (10)	110/911 (12)	107/858 (12)	3/53 (3)	0.27 (0.07 to 1.09)	-9.85
**LAC**						
Guyana	209/3,182 (7)	116/1,556 (7)	90/1,278 (7)	26/278 (9)	1.36 (0.67 to 2.76)	1.86

Child labour among boys

Country	All children n/N (%)	Girls n/N (%)	No disability n/N (%)	With disability n/N (%)	Age-adj percentage ratio (95% CI)	Age-adj percentage difference
Suriname	121/3,867 (3)	73/1,944 (4)	55/1,650 (3)	18/294 (8)	2.19 (1.34 to 3.59)	4.83
**MENA**						
Algeria	235/16,243 (1)	165/8,512 (2)	124/6,511 (2)	41/2,001 (3)	1.31 (0.81 to 2.12)	0.75
Iraq	449/15,486 (3)	340/8,075 (4)	237/6,115 (4)	103/1,960 (5)	1.36 (0.94 to 1.98)	1.44
State of Palestine	323/5,251 (6)	246/2,739 (9)	214/2,263 (9)	32/476 (7)	0.76 (0.52 to 1.11)	-2.34
**SA**						
Bangladesh	2,178/39,265 (6)	1,593/20,205 (8)	1,438/18,534 (8)	155/1,671 (10)	1.29 (1.08 to 1.54)	2.45
**SSA**						
CAR	1,431/5,972 (24)	675/3,002 (22)	453/2,087 (22)	222/915 (24)	1.12 (0.91 to 1.37)	2.68
Chad	4,722/14,608 (32)	2,364/7,207 (33)	1,676/4,920 (34)	688/2,287 (30)	0.90 (0.83 to 0.97)	-3.78
DRC Congo	1,843/13,941 (13)	826/6,901 (12)	621/5,297 (12)	205/1,604 (11)	0.91 (0.66 to 1.25)	-0.99
Ghana	1,469/8,927 (16)	716/4,551 (16)	546/3,606 (15)	170/945 (19)	1.24 (0.94 to 1.65)	3.60
Guinea-Bissau	959/5,835 (16)	496/2,850 (17)	408/2,478 (16)	88/372 (20)	1.18 (0.88 to 1.58)	3.07
Lesotho	755/4,918 (15)	526/2,479 (21)	483/2,278 (21)	43/201 (22)	1.05 (0.78 to 1.42)	1.00
Madagascar	3,745/11,915 (31)	2,069/5,949 (35)	1,781/5,067 (35)	288/882 (34)	0.98 (0.87 to 1.09)	-0.86
Sao Tome and Principe	182/2,165 (8)	83/1,072 (8)	66/841 (8)	17/231 (7)	0.93 (0.49 to 1.77)	-0.49
Sierra Leone	2,776/10,863 (26)	1,418/5,333 (27)	1,101/4,009 (27)	317/1,324 (23)	0.82 (0.72 to 0.94)	-4.61
The Gambia	924/5,649 (16)	434/2,538 (17)	386/2,275 (17)	48/263 (18)	1.06 (0.75 to 1.49)	0.90
Togo	1,594/4,917 (32)	801/2,469 (32)	631/1,968 (32)	170/501 (36)	1.10 (0.90 to 1.35)	3.65
Zimbabwe	1,672/7,005 (24)	1,062/3,622 (29)	940/3,253 (29)	122/369 (34)	1.16 (0.99 to 1.35)	4.99

Hazardous labour among girls

Country	All children n/N (%)	Girls n/N (%)	No disability n/N (%)	With disability n/N (%)	Age-adj percentage ratio (95% CI)	Age-adj percentage difference
**EAP**						
Mongolia	610/7,331 (8)	202/3,545 (6)	189/3,326 (6)	13/219 (4)	0.78 (0.37 to 1.64)	-1.26
Tonga	489/1,621 (30)	157/776 (20)	143/696 (21)	14/80 (25)	1.29 (0.57 to 2.93)	4.53
**ECA**						
Kosovo	122/2,347 (5)	17/1,060 (2)	14/968 (1)	3/92 (3)	2.14 (0.54 to 8.52)	1.83
Kyrgyzstan	419/3,889 (11)	113/1,835 (6)	97/1,695 (6)	16/140 (10)	1.69 (0.96 to 2.96)	4.35
North Macedonia	46/1,428 (3)	5/708 (1)	5/637 (1)	0/71 (0)	-	-0.53
Serbia	37/1,726 (2)	6/815 (1)	5/790 (1)	1/25 (3)	2.54 (0.28 to 22.93)	2.18
**LAC**						
Guyana	324/3,186 (10)	127/1,624 (8)	88/1,346 (7)	39/278 (9)	1.47 (0.87 to 2.50)	2.51
Suriname	101/3,873 (3)	29/1,931 (2)	23/1,680 (1)	6/251 (4)	2.79 (1.00 to 7.79)	2.27
**MENA**						
Algeria	348/16,374 (2)	59/7,790 (1)	45/6,357 (1)	14/1,433 (1)	1.63 (0.80 to 3.34)	0.54
Iraq	824/15,523 (5)	173/7,421 (2)	133/6,004 (2)	40/1,417 (2)	1.11 (0.83 to 1.47)	0.26
State of Palestine	274/5,264 (5)	32/2,520 (1)	23/2,211 (1)	9/309 (4)	3.68 (1.20 to 11.30)	2.82
**SA**						
Bangladesh	3,116/39,284 (8)	684/19,068 (4)	614/17,754 (3)	70/1,314 (5)	1.48 (1.06 to 2.07)	1.68
**SSA**						
CAR	1,753/5,960 (29)	894/2,967 (30)	569/2,030 (28)	325/937 (36)	1.29 (1.10 to 1.53)	8.40
Chad	5,853/14,629 (40)	2,743/7,409 (37)	1,930/5,152 (37)	813/2,257 (38)	1.02 (0.95 to 1.09)	0.79
DRC Congo	2,309/13,946 (17)	1,208/7,046 (17)	907/5,466 (17)	301/1,580 (19)	1.21 (0.93 to 1.57)	2.43
Ghana	1,761/8,925 (20)	807/4,375 (18)	618/3,504 (18)	189/871 (27)	1.50 (1.18 to 1.90)	8.98
Guinea-Bissau	1,629/5,836 (28)	833/2,985 (28)	649/2,564 (25)	184/421 (49)	1.95 (1.75 to 2.16)	23.77
Lesotho	611/4,921 (12)	162/2,444 (7)	141/2,256 (6)	21/188 (11)	1.67 (0.92 to 3.03)	4.34

Hazardous labour among girls

Country	All children n/N (%)	Girls n/N (%)	No disability n/N (%)	With disability n/N (%)	Age-adj percentage ratio (95% CI)	Age-adj percentage difference
Madagascar	3,749/11,915 (31)	1,647/5,968 (28)	1,429/5,150 (28)	218/818 (29)	1.05 (0.88 to 1.25)	1.39
Sao Tome and Principe	312/2,167 (14)	148/1,090 (14)	127/853 (15)	21/237 (8)	0.56 (0.33 to 0.95)	-6.99
Sierra Leone	3,691/10,866 (34)	1,793/5,529 (32)	1,363/4,260 (32)	430/1,269 (38)	1.23 (1.07 to 1.42)	6.39
The Gambia	921/5,641 (16)	475/3,108 (15)	430/2,833 (15)	45/275 (16)	1.04 (0.71 to 1.51)	0.48
Togo	1,489/4,917 (30)	655/2,447 (27)	479/1,946 (25)	176/501 (38)	1.54 (1.29 to 1.83)	13.56
Zimbabwe	835/7,006 (12)	324/3,385 (10)	298/3,100 (10)	26/285 (7)	0.77 (0.43 to 1.39)	-2.42

Hazardous labour among boys

Country	All children n/N (%)	Girls n/N (%)	No disability n/N (%)	With disability n/N (%)	Age-adj percentage ratio (95% CI)	Age-adj percentage difference
**EAP**						
Mongolia	610/7,331 (8)	408/3,786 (11)	383/3,562 (11)	25/224 (15)	1.43 (0.84 to 2.43)	4.09
Tonga	489/1,621 (30)	332/845 (39)	294/759 (39)	38/86 (53)	1.37 (0.93 to 2.02)	13.80
**ECA**						
Kosovo	122/2,347 (5)	105/1,287 (8)	96/1,186 (8)	9/101 (8)	1.00 (0.55 to 1.81)	-0.00
Kyrgyzstan	419/3,889 (11)	306/2,054 (15)	275/1,897 (14)	31/157 (21)	1.43 (0.94 to 2.17)	6.95
North Macedonia	46/1,428 (3)	41/720 (6)	36/634 (6)	5/86 (7)	1.28 (0.45 to 3.58)	1.47
Serbia	37/1,726 (2)	31/911 (3)	27/858 (3)	4/53 (4)	1.14 (0.31 to 4.26)	0.42
**LAC**						
Guyana	324/3,186 (10)	197/1,562 (13)	147/1,282 (11)	50/280 (18)	1.74 (1.15 to 2.65)	6.33
Suriname	101/3,873 (3)	72/1,942 (4)	54/1,648 (3)	18/294 (9)	3.12 (1.59 to 6.11)	6.22
**MENA**						
Algeria	348/16,374 (2)	289/8,584 (3)	210/6,562 (3)	79/2,022 (4)	1.09 (0.73 to 1.63)	0.34
Iraq	824/15,523 (5)	651/8,102 (8)	476/6,141 (8)	175/1,961 (10)	1.24 (1.00 to 1.53)	2.11
State of Palestine	274/5,264 (5)	242/2,744 (9)	200/2,269 (9)	42/475 (11)	1.31 (0.96 to 1.78)	2.53

Hazardous labour among boys

Country	All children n/N (%)	Girls n/N (%)	No disability n/N (%)	With disability n/N (%)	Age-adj percentage ratio (95% CI)	Age-adj percentage difference
**SA**						
Bangladesh	3,116/39,284 (8)	2,432/20,216 (12)	2,204/18,544 (12)	228/1,672 (15)	1.28 (1.14 to 1.44)	3.31
**SSA**						
CAR	1,753/5,960 (29)	859/2,993 (29)	552/2,080 (27)	307/913 (37)	1.40 (1.21 to 1.62)	10.84
Chad	5,853/14,629 (40)	3,110/7,220 (43)	2,157/4,928 (44)	953/2,292 (45)	1.02 (0.96 to 1.07)	0.79
DRC Congo	2,309/13,946 (17)	1,101/6,900 (16)	849/5,296 (16)	252/1,604 (17)	1.05 (0.75 to 1.47)	0.64
Ghana	1,761/8,925 (20)	954/4,550 (21)	716/3,606 (20)	238/944 (30)	1.50 (1.27 to 1.78)	9.98
Guinea-Bissau	1,629/5,836 (28)	796/2,851 (28)	636/2,478 (26)	160/373 (45)	1.80 (1.54 to 2.10)	19.23
Lesotho	611/4,921 (12)	449/2,477 (18)	411/2,276 (18)	38/201 (21)	1.16 (0.77 to 1.76)	2.82
Madagascar	3,749/11,915 (31)	2,102/5,947 (35)	1,803/5,065 (36)	299/882 (37)	1.04 (0.92 to 1.18)	1.52
Sao Tome and Principe	312/2,167 (14)	164/1,077 (15)	130/847 (15)	34/230 (18)	1.21 (0.73 to 2.01)	2.83
Sierra Leone	3,691/10,866 (34)	1,898/5,337 (36)	1,421/4,006 (35)	477/1,331 (38)	1.07 (0.92 to 1.24)	2.16
The Gambia	921/5,641 (16)	446/2,533 (18)	409/2,271 (18)	37/262 (11)	0.61 (0.42 to 0.89)	-6.68
Togo	1,489/4,917 (30)	834/2,470 (34)	639/1,968 (32)	195/502 (39)	1.18 (0.98 to 1.41)	6.17
Zimbabwe	835/7,006 (12)	511/3,621 (14)	436/3,252 (13)	75/369 (18)	1.32 (1.05 to 1.67)	4.87

Fig A: Relative inequities in birth registration, birth certification, child labour, and violent discipline by disability status

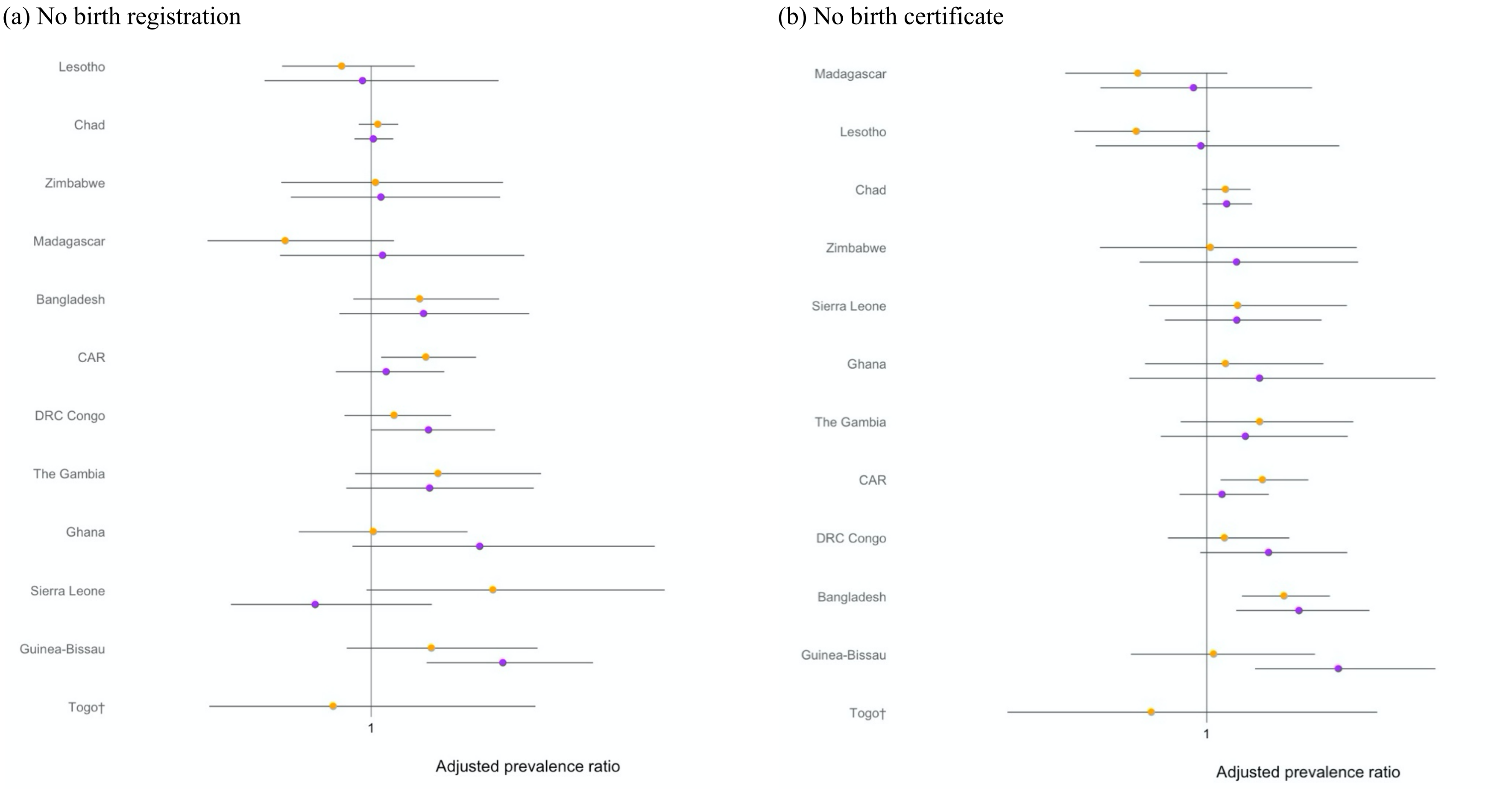

Figure shows country specific age adjusted prevalence ratios among girls (purple) and boys (orange). † indicates small cell sizes (<30 participants). Figure includes the 12 countries that have not achieved near-universal birth registration.

Fig B: Relative inequities in birth registration, birth certification, child labour, and violent discipline by disability status (contd.)

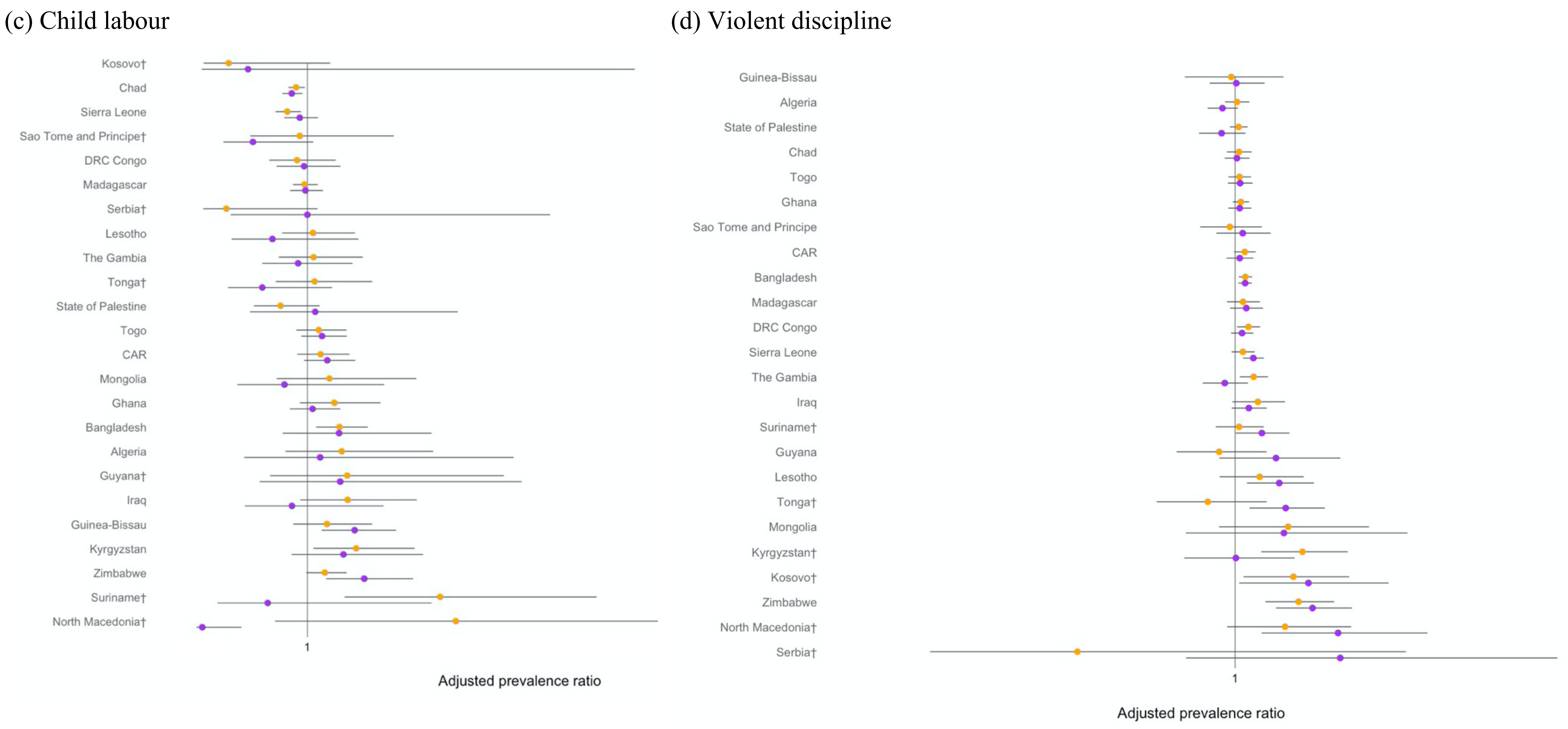

Figure shows country specific age adjusted prevalence ratios among girls (purple) and boys (orange). † indicates small cell sizes (<30 participants).
